# Health Care Support Worker Status, Health Behaviors, Mental Health, and Preventive Health Care Use

**DOI:** 10.1001/jamanetworkopen.2023.48578

**Published:** 2023-12-26

**Authors:** Jin Jun, Heather L. Tubbs-Cooley, Matthew A. Davis

**Affiliations:** 1 The Center for Healthy Aging, Self-Management and Complex Care, The Ohio State University College of Nursing, Columbus; 2 The Martha S. Pitzer Center for Women, Children and Youth, The Ohio State University College of Nursing, Columbus; 3 Department of Systems, Populations and Leadership, University of Michigan School of Nursing, Ann Arbor; 4 Department of Learning Health Sciences, University of Michigan Medical School, Ann Arbor

## Abstract

This cross-sectional study compares the health behaviors, mental health status, and preventive health care usage of health care support workers (HSWs) with clinicians and the general population.

## Introduction

Health care support workers (HSWs), such as nursing assistants and home health aides, represent a rapidly growing segment of the health care workforce, expected to reach 4.6 million by 2031.^1,2^ Despite their crucial role in providing hands-on essential services, they are among the lowest paid, earning a median annual wage of $34 500.^1^ HSWs assume physical risks in their roles and work long hours,^3,4,5,6^ but little is known about their health. Therefore, we examined and compared health behaviors, mental health status, and preventive health care usage of HSWs with clinicians and the general population.

## Methods

We conducted a cross-sectional study using the 2014 to 2018 National Health Interview Survey (NHIS) Sample Adult Files data. The study followed the STROBE reporting guideline and received exemption from review and the requirement for informed consent, because this was a secondary analysis of publicly available data, from the Ohio State University institutional review board. Two occupational classifications were considered: HSWs and clinicians (health diagnosing and treating health care practitioners), while all other occupations represented the general population of working adults (eAppendix in Supplement 1). Outcomes of interest were health behaviors (alcohol use, smoking, and sleep), mental health status (depression and anxiety screening), and preventive health care usage in the past 12 months (availability of usual places of sick or routine care; completion of mammogram, colonoscopy, Papanicolaou (Pap) test, and influenza vaccine). NHIS data were appended to represent a single year and a complex survey design to generate national estimates. Descriptive analyses compared groups using *t* tests and χ^2^ tests, while logistic models were used to calculate associations, adjusting for age, sex, race and ethnicity, family income, and marital status. All analyses were based on complete case analysis using Stata version 17.0 (StataCorp). The statistical significance level was set at a critical α level of .05 and was 2-sided.

## Results

**Figure 1.**
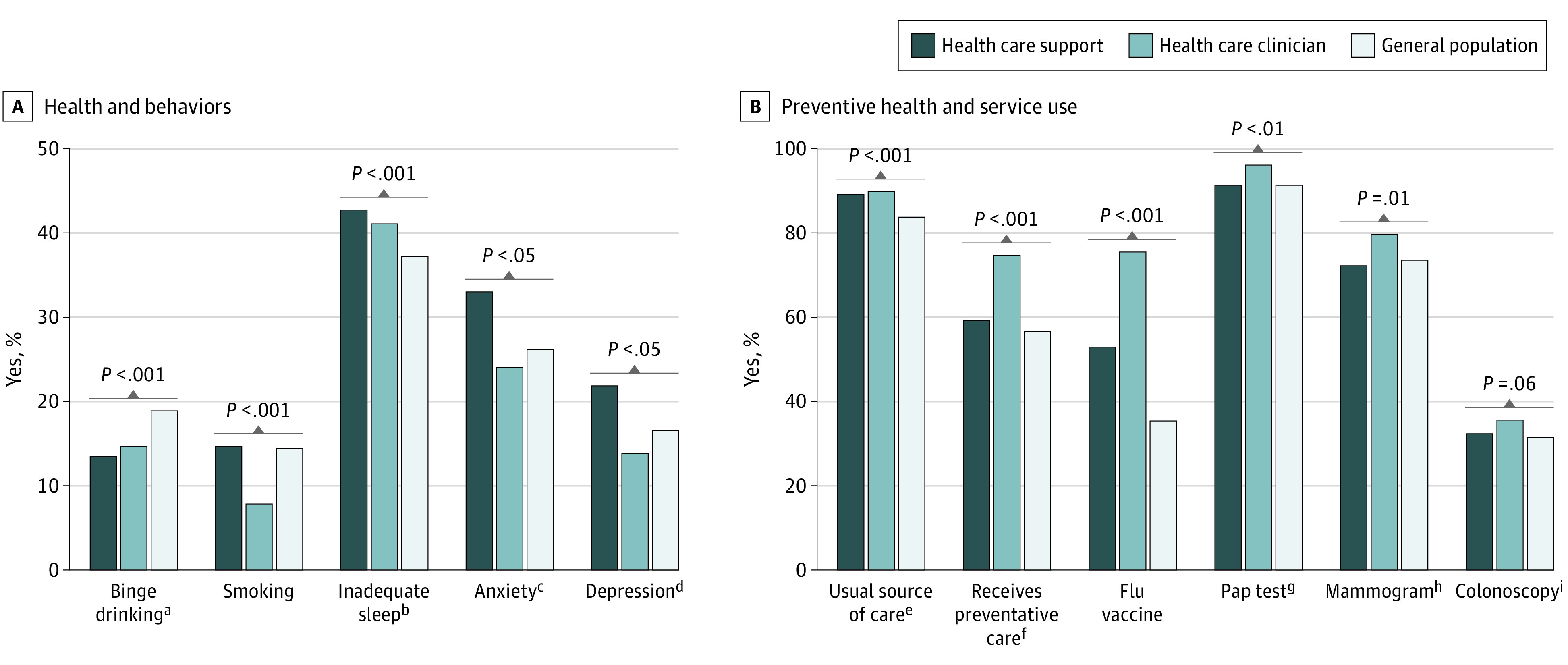
Health, Behaviors, and Preventive Health Service Use Among Health Care Support Workers, Clinicians, and the General Population All analyses were weighted to represent the US population and adjusted for sex, race and ethnicity, level of education, household income level, marital status, and age (continuous). The general population represents US employed adults (age ≥18 years). ^a^Binge drinking was defined as having more than 5 (for men) or 4 (for women) drinks or any alcoholic beverage during the past 30 days (based on the annual National Survey on Drug Use and Health 2019 binge drinking item). ^b^Inadequate sleep was defined as less than 7 hours of sleep, based on the American Academy of Sleep Medicine and Sleep Research Society, 2015. ^c^Anxiety screening was defined as self-reported anxiety experienced on some or most days, based on General Anxiety Disorder items (equivalent to a General Anxiety Disorder score >2). ^d^Depression was defined as self-reported depression experienced on some or most days based on the Patient Health Questionnaire items (equivalent to a Patient Health Questionnaire score >2). ^e^Usual source of care was defined as self-report of having a usual place for sick and routine or preventive medical care. ^f^Receives preventive care defined as self-report of receiving preventive care in the last 12 months. ^g^Papanicolaou (Pap) test defined as having received a Pap test in the last 12 months, restricted to female sex. ^h^Mammogram defined as having received a mammogram in the past 12 months, restricted to female sex and age 50 years and older. ^i^Colonoscopy defined as having received colon cancer screening in the past 12 months, restricted to participants age 45 years and older.

There were an estimated 2.3 million HSWs in the United States. The mean (SE) age of HSWs was 40.2 (0.6) years, 88.8% were female, and 25.0% were Black. HSWs had higher rates of inadequate sleep (42.7% vs 41.1% of clinicians and 37.2% of the general population; *P* = .004), anxiety (33.0% vs 24.0% and 26.2%), and depression (19.1% vs 13.8% and 16.5%) (*P* < .001 for both). HSWs were less likely than clinicians to receive preventive care (59.2% vs 74.6%) or an influenza vaccine in the last 12 months (52.8% vs 47.2%) (*P* < .001 for both) (Figure 1).

**Figure 2.**
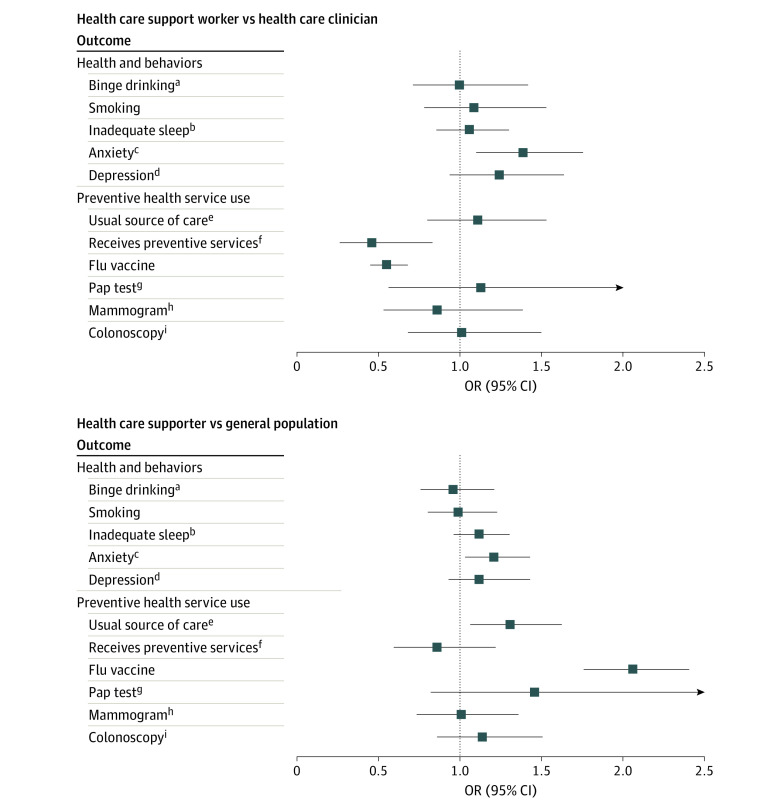
Association Between Health Care Support Worker Status and Health, Behaviors, and Preventive Health Service Use All analyses weighted to represent the US population and adjusted for sex, race and ethnicity, level of education, household income level, marital status, and age (continuous). General population represents US employed adults (age ≥18 years). OR indicates odds ratio. ^a^Binge drinking was defined as having more than 5 (for men) or 4 (for women) drinks or any alcoholic beverage during the past 30 days, based on the annual National Survey on Drug Use and Health 2019 binge drinking item. ^b^Inadequate sleep was defined as less than 7 hours of sleep, based on the American Academy of Sleep Medicine and Sleep Research Society, 2015. ^c^Anxiety defined as self-reported anxiety experienced on some or most days based on General Anxiety Disorder items (equivalent to General Anxiety Disorder score >2). ^d^Depression defined as self-reported depression experienced on some or most days based on Patient Health Questionnaire items (equivalent to Patient Health Questionnaire score >2). ^e^Usual source of care was defined as self-report of having a usual place for sick and routine or preventive medical care. ^f^Receives preventive care defined as self-report of receiving preventive care in the last 12 months. ^g^Papanicolaou (Pap) test defined as having received a Pap test in the last 12 months, restricted to female sex. ^h^Mammogram defined as having received a mammogram in the past 12 months, restricted to female sex and age 50 years and older. ^i^Colonoscopy defined as having received colon cancer screening in the past 12 months, restricted to participants age 45 years and older.

In adjusted analyses (Figure 2), HSWs were 39% more likely to have anxiety compared with clinicians (adjusted odds ratio [AOR], 1.39; 95% CI, 1.10-1.76) and 21% more likely compared with the general population (AOR, 1.21; 95% CI, 1.03-1.43). HSWs were also less likely than clinicians to receive preventive care (AOR, 0.46; 95% CI, 0.26-0.83) or the influenza vaccine (AOR, 0.55; 95% CI, 0.45-0.68). However, HSWs were more likely than the general population to have a usual place for illness and/or routine care (AOR, 1.31; 95% CI, 1.06-1.63) and more likely to receive the influenza vaccine (AOR, 2.06; 95% CI, 1.76-2.41).

## Discussion

We found that HSWs experienced more depression and anxiety than clinicians (a group already known to be at high risk for mental health disorders) and the general population. The risks of anxiety persisted among HSWs compared with both groups when adjusted for socioeconomic factors. Furthermore, we found evidence that HSWs were less likely than clinicians to receive preventive services. Although we could not determine the degree to which work schedules or how the types of health care benefits affect the health of HSWs, our finding highlights the critical need to develop targeted interventions to meet the needs of this understudied population. The limitations of the study are the potential for residual confounding, lower specificity in the screening questions, and the inability to determine causal relationships.

## Notes

Supplement 1. **eAppendix.** Occupation Classification Codes Used in the National Health Interview Survey Data, 2010-2018

Supplement 2.
Data Sharing Statement
