# Supplementary material for: Health Care Support Worker Status, Health Behaviors, Mental Health, and Preventive Health Care Use
Source: JAMA Netw Open. 2023 Dec 26;6(12):e2348578. doi: 10.1001/jamanetworkopen.2023.48578 (PMC10751586; doi:10.1001/jamanetworkopen.2023.48578)
Supplement: Supplement 1. — eAppendix. Occupation Classification Codes Used in the National Health Interview Survey Data, 2010-2018 [file jamanetwopen-e2348578-s001.pdf]

Supplemental Online Content

Jun J, Tubbs Cooley HL, Davis MA. Health care support worker status, health behaviors, mental health, and preventive healthcare use. *JAMA Netw Open*. 2023;6(12):e2348578. doi:10.1001/jamanetworkopen.2023.48578

eAppendix. Occupation Classification Codes Used in the National Health Interview Survey Data, 2010-2018

This supplemental material has been provided by the authors to give readers additional information about their work.

© 2023 Jun J et al. *JAMA Network Open.*

eAppendix. Occupation Classification Codes Used in the National Health Interview Survey Data, 2010-2018

National Health Interview Survey (NHIS) is conducted annually by the Centers for Disease Control and Prevention to produce nationally representative information on the health of the U.S. civilian, noninstitutionalized population, using a multistage clustered sample design. This supplement page provides the occupational classification code (OCC) system used in the NHIS to indicate participants' occupations. This OCC system was used from 2010-2018 and was based on the U.S. Census 4-digit occupation codes informed by the Standard Occupation Classification (SOC).

Participants' occupations have been collected as a part of the NHIS annual sociodemographic section since 1997, using the occupation codes based on the SOC. To protect the confidentiality of respondents, NHIS staff removed the detailed 3-digit and 4-digit codes from the public use NHIS files and replaced them with 2-digit recodes for occupation classification. The occupational coding schemes used in the NHIS have changed over the years several times, reflecting the dynamic nature and changes in occupations and industries. However, they also limit the comparability of occupations or industries across NHIS surveys. In 2019, the content and structure of NHIS underwent a significant update, to improve the measurement of covered health topics and to reduce respondents' burden. During this change, the occupational codes were not included in 2019 but resumed in 2020 using the updated 2018 SOC.

eTable. Occupational codes used to identify Healthcare Support Workers and Healthcare Clinicians

2010-2018 Occupation Classification Code	2010-2018 Occupation Classification Categories	2010 Census Codes	2010 Census Occupational Code Description
**Healthcare Clinician and Technician Occupations**			
29	Health diagnosing and treating practitioners	3000, 3020, 3030, 3040, 3050, 3060, 3110, 3120, 3140, 3150, 3160, 3200, 3210, 3220, 3230, 3235, 3245, 3250, 3255, 3256, 3257, 3260	Chiropractors, dentists, dietitians & nutritionists, optometrists, pharmacists, physicians and surgeons, physician assistants, podiatrists, audiologists, occupational therapists, physical therapists, radiation therapists, recreational therapists, respiratory therapists, speech-language therapists, exercise

© 2023 Jun J et al. *JAMA Network Open.*

			physiologists, all other therapists, veterinarians, registered nurses, nurse anesthetists, nurse midwives, nurse practitioners, & all other health diagnosing and treating practitioners,
30	Health technologists and technicians	3300, 3310, 3320, 3400, 3420, 3500, 3510, 3520, 3535	Clinical laboratory technologists & technicians, dental hygienists, emergency medical technicians and paramedics, licensed practical and licensed vocational nurses, medical records and health information technicians, dispensing opticians
31	Other healthcare practitioners and technical occupations	3540	Miscellaneous health technologists and technicians
**Healthcare Support Occupations**			
32	Nursing, psychiatric, and home health aides	3600	Nursing, psychiatrics, and home health aides,
33	Occupational and physical therapist assistants and aides	3610, 3620	Occupational and physical therapist assistants and aides, and
34	Other healthcare support occupations	3630, 3640, 3645, 3646, 3647, 3648, 3649, 3535	Message therapists, dental assistants, medical assistants, medical transcriptionists, pharmacy technicians and aides, veterinary assistants and laboratory animal caretakers, phlebotomists, medical equipment preparers, all other healthcare support workers)

© 2023 Jun J et al. *JAMA Network Open.*

The restricted variables were accessed through the CDC's National Center for Health Statistics Research Data Center. Only the publicly available dataset was used in this study.

Sources:
Lynn A. Blewett, Julia A. Rivera Drew, Miriam L. King, Kari C.W. Williams, Natalie Del Ponte and Pat Convey. IPUMS Health Surveys: National Health Interview Survey, Version 7.2 [dataset]. Minneapolis, MN: IPUMS, 2022. https://doi.org/10.18128/D070.V7.2 Accessed on https://nhis.ipums.org/

Centers for Disease Control and Prevention. National Center for Health Statistics. https://www.cdc.gov/nchs/. July 13, 2023.

© 2023 Jun J et al. *JAMA Network Open.*

© 2023 Jun J et al. *JAMA Network Open.*
