# Supplementary material for: Health Care Support Worker Status, Health Behaviors, Mental Health, and Preventive Health Care Use
Source: JAMA Netw Open. 2023 Dec 26;6(12):e2348578. doi: 10.1001/jamanetworkopen.2023.48578 (PMC10751586; doi:10.1001/jamanetworkopen.2023.48578)
Supplement: Supplement 2. — Data Sharing Statement [file jamanetwopen-e2348578-s002.pdf]

Data Sharing Statement

Jun. Health Care Support Worker Status, Health Behaviors, Mental Health, and Preventive Healthcare Use. *JAMA Netw Open*. Published December 20, 2023.
doi:10.1001/jamanetworkopen.2023.48578

Data
Data available: No

Additional Information
Explanation for why data not available: The data used in this study is publically available on the National Center for Health Statistics.
